# PoPoolation DB: a user-friendly web-based database for the retrieval of natural polymorphisms in Drosophila

**DOI:** 10.1186/1471-2156-12-27

**Published:** 2011-03-02

**Authors:** Ram Vinay Pandey, Robert Kofler, Pablo Orozco-terWengel, Viola Nolte, Christian Schlötterer

**Affiliations:** 1 Institut für Populationsgenetik, Vetmeduni Vienna, Veterinärplatz 1, Vienna, Austria

## Abstract

**Background:**

The enormous potential of natural variation for the functional characterization of genes has been neglected for a long time. Only since recently, functional geneticists are starting to account for natural variation in their analyses. With the new sequencing technologies it has become feasible to collect sequence information for multiple individuals on a genomic scale. In particular sequencing pooled DNA samples has been shown to provide a cost-effective approach for characterizing variation in natural populations. While a range of software tools have been developed for mapping these reads onto a reference genome and extracting SNPs, linking this information to population genetic estimators and functional information still poses a major challenge to many researchers.

**Results:**

We developed PoPoolation DB a user-friendly integrated database. Popoolation DB links variation in natural populations with functional information, allowing a wide range of researchers to take advantage of population genetic data. PoPoolation DB provides the user with population genetic parameters (Watterson's *θ *or Tajima's *π*), Tajima's D, SNPs, allele frequencies and indels in regions of interest. The database can be queried by gene name, chromosomal position, or a user-provided query sequence or GTF file. We anticipate that PoPoolation DB will be a highly versatile tool for functional geneticists as well as evolutionary biologists.

**Conclusions:**

PoPoolation DB, available at http://www.popoolation.at/pgt, provides an integrated platform for researchers to investigate natural polymorphism and associated functional annotations from UCSC and Flybase genome browsers, population genetic estimators and RNA-seq information.

## Background

The functional implications of natural variation has been a long-standing interest of evolutionary biologists. Nevertheless, only recently, functional biologists are starting to recognize that natural variation could also provide important insights into the function of genes. Naturally occurring alleles could be viewed as the outcome of a large-scale mutagenesis experiment focusing on mutations with a smaller effect and/or different functionality. Some functional studies have successfully accounted for natural variation in their analyses [1,2]. The new sequencing technologies provide an unprecedented opportunity to collect sequence information on a genomic scale for a large number of individuals. In particular when pools of genomic DNA are sequenced, it has become feasible to collect population variation data on a genomic scale within the budget of a typical research grant. In the wake of the new sequencing technologies, also new software has been developed for mapping short sequence reads onto a reference genome and extracting SNPs. Nevertheless, linking this information with functional information (exons, introns, transcription levels etc.) still poses a major challenge to many researchers. Furthermore, it is difficult for non-experts to extract population genetic estimators from the vast amounts of sequencing data.

We developed PoPoolation DB http://www.popoolation.at/pgt/ a user-friendly and integrated resource to link variation in natural populations with functional information, allowing a wide range of researchers to take advantage of population genetic data.

PoPoolation DB allows the retrieval of polymorphism data from pooled NGS sequence data using new statistical approaches to obtain population genetic parameters from pooled data [3]. PoPoolation DB can be queried by gene name, chromosomal position, or a user-provided query sequence or GTF file.

## Construction and content

### Database and web interface development

**Figure 1:**
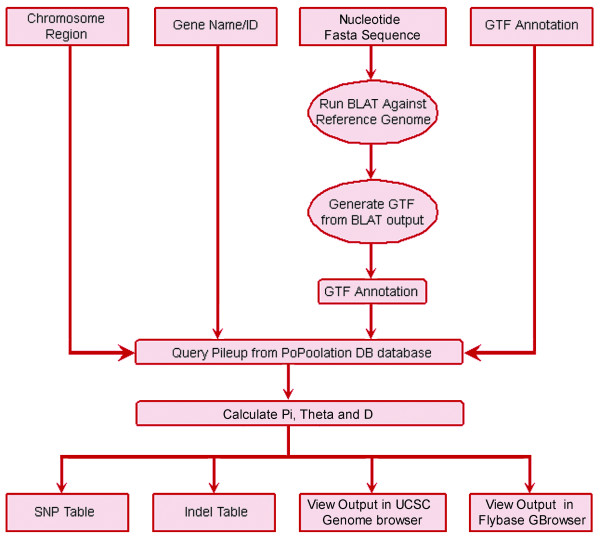
**The schema and architecture of PoPoolation DB database**. The data flow in PoPoolation DB from query page to result page with different query types (Query by region, Query by gene, Query by DNA fasta sequence and Query by GTF).

The PoPoolation DB database was developed in MySQL 5.1.The PoPoolation DB web interface was developed using the CGI.pm, DBI.pm and DBD::mysql.pm modules of Perl (5.8.8), and runs on an Apache (2.0.53) web server. PoPoolation DB is a relational database that contains information about short sequence reads mapped to a reference genome. Graphical display of genetic variation parameters (Watterson's θ or Tajima's π) and Tajimas' D [4] for genomic region of interest was incorporated in the UCSC genome browser, FlyBase Genome browser and in Flybase RNA-seq browser. Hence, population genetic parameters are visualized along with functional annotation available in these browsers. Figure 1 provides an overview of the architecture and features of the PoPoolation DB database.

### Fly samples and Sequencing

Genomic DNA was extracted from a pool of flies consisting of five females from each of 113 isofemale lines collected 2008 in Povoa de Varzim (Northern Portugal). DNA was extracted from homogenized pooled flies with the Qiagen DNeasy Blood and Tissue Kit (Qiagen, Hilden, Germany). Sequencing followed standard Illumina protocols using Paired End Cluster Generation Kit v2 and sequencing Kits v3 on a Genome Analyzer IIx. Image analysis was performed with the Firecrest, Bustard and Gerald modules of the Illumina pipeline v. 1.4.

### Mapping

Reads were trimmed on both ends for base quality 20 and minimum sequence length of 40 base pairs. The trimmed reads were mapped to the *D. melanogaster *reference genome (v. 5.18) using the global alignment algorithm implemented in bwa [5,6]. Mapping parameters were: seeding disabled, error rate of 1% (-n 0.01), maximum of two gap openings (-o 2) and gap extension of a maximum of 12 bases (-e 12, -d 12). Paired-end reads were further processed with the sampe module in bwa enabling the Smith-Waterman algorithm for the unmapped mate and allowing a maximum of 500 bp between the read pairs. Reads left without a pair were removed. Only reads with a minimum mapping quality of 20 were used. The final pileup output was stored in PoPoolation DB.

### Data source

In order to link natural polymorphism with genomic annotation of reference genome, we have downloaded *Drosophila melanogaster *reference genome Fasta sequences (release 5) and GFF3 annotation file (v. 5.32) from FlyBase database [7].

## Utility and Discussion

### Query interface

**Figure 2:**
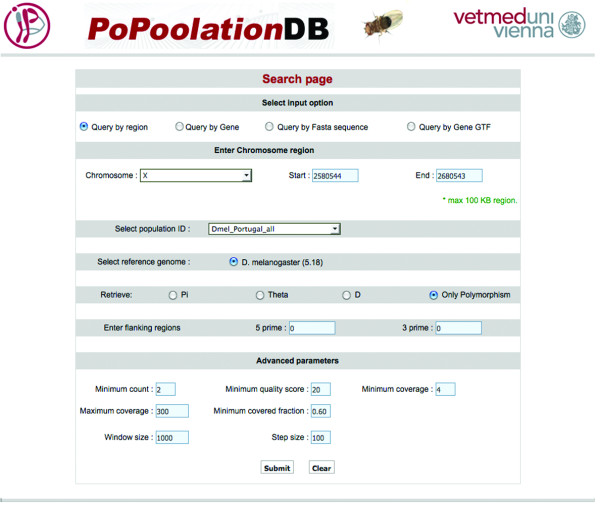
**PoPoolation DB query page**. The query interface for PoPoolation DB shows different query options and parameters that can be used to search natural polymorphism and population genetic estimators.

PoPoolation DB offers a very powerful and flexible query interface (Figure 2). The query interface and web interface of PoPoolation DB are able to handle the integration of further data (more population and species). Popoolation DB allows user to query this database by 1) chromosomal region 2) gene 3) DNA fasta sequence and 4) GTF.

### Web interface

**Figure 3:**
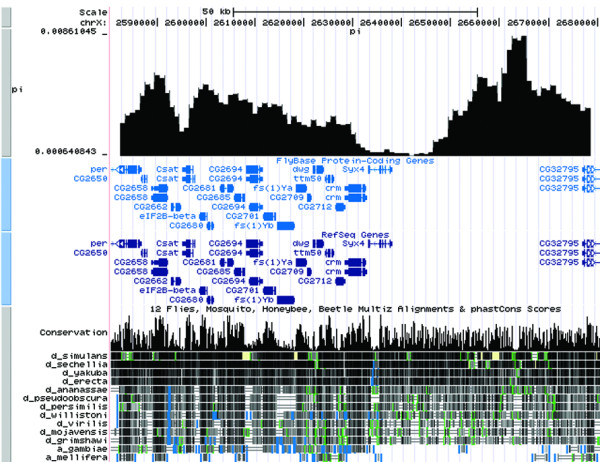
**An example output of the θπ track visualization in UCSC genome browser**. An example output of sliding window analysis of Tajima's π of a Portuguese *D*. *melanogaster *population on chromosome X: 2044135-2053016 displayed in the linked UCSC genome browser. The pronounced drop in variability around the gene *crm *has been previously described, suggesting that at least one favorable mutation has recently spread in cosmopolitan *D*. *melanogaster *populations [12].

**Figure 4:**
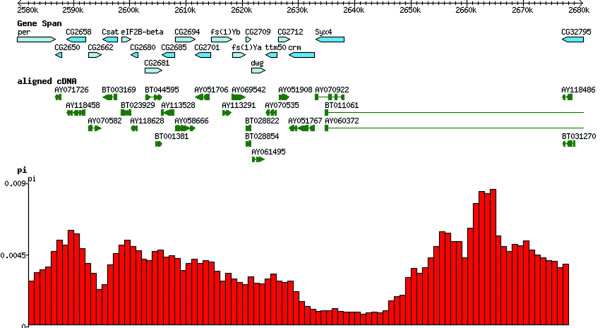
**An example output of the θπ track visualization in FlyBase genome browser**. An example output of sliding window analysis of Tajima's π in a Portuguese *D*. *melanogaster *population displayed in the Flybase genome browser. The displayed region corresponds to position 2044135-2053016 on the X chromosome. The pronounced drop in variability around the gene *crm* has been described previously, suggesting that at least one favorable mutation has recently spread in cosmopolitan *D*. *melanogaster *populations [12].

**Figure 5:**
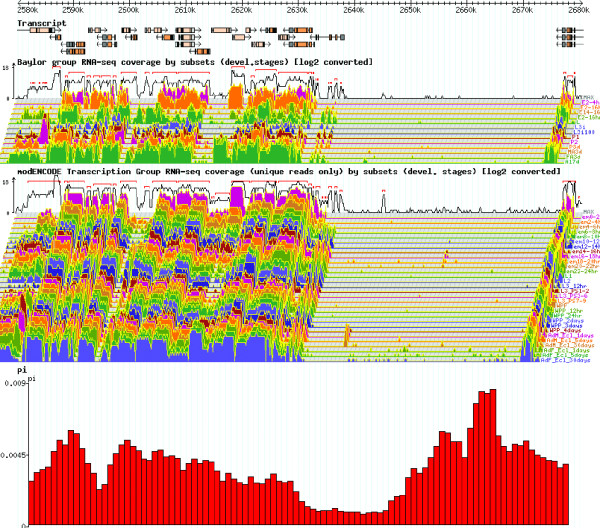
**An example output of the θπ track visualization in FlyBase RNA-Seq browser**. An example output of the sliding window analysis of Tajima's π as shown in Figure 4 displayed with the RNAseq profiles of different developmental stages and cell line expression data of D. *melanogaster *stored in Flybase.

**Figure 6:**
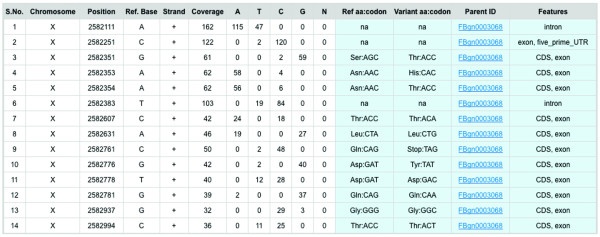
**An example output of SNP and Codon information table in PoPoolation DB**. An example output of SNP and codon information table. The table provides information about the chromosomal location of the SNP, the character state in the reference genome, the number of reads, the sequence feature (intron, UTR, coding sequence), and a link to the Flybase Gene ID. For polymorphisms in the coding sequence the corresponding amino acid is also displayed, allowing the distinction between synonymous and non-synonymous SNPs).

**Figure 7:**
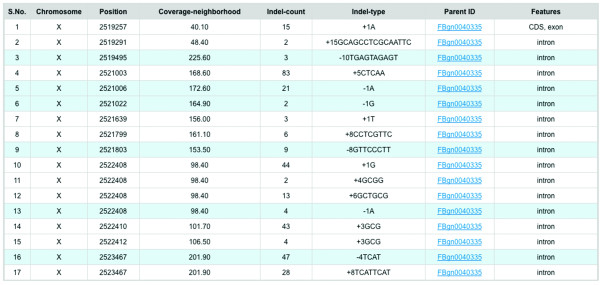
**An example output of Indel information table in PoPoolation DB**. An example output of Indel information table. The table provides the chromosomal location, the average number of reads covering 10 bp flanking the indel, the number of different indels at the same position, the frequency of the indel in the population and sequence information about the indel, the sequence feature (intron, UTR, coding sequence), and a link to the Flybase Gene ID.

PoPoolation DB provides a user-friendly web interface that allows the retrieval of polymorphism data from pooled NGS sequence data using new statistical approaches to obtain population genetic parameters from pooled data [3]. Figure 1 shows the schema and architecture of PoPoolation DB. Users can query PoPoolation DB using gene names (e.g. *crm*), gene IDs (e.g. CG2714), genomic regions (e.g. X:2,628,277..2,632,810), DNA sequences and standard GTF files (Figure 1, Figure 2). The output consists of a graphical representation of a population variation parameter (Watterson's *θ *or Tajima's *π*) or Tajimas' D, a widely used indicator variable for the identification of natural selection (Figure 3). To link these population polymorphisms to functional information, the user may choose to display the graphical output in the UCSC Genome Browser [8] (Figure 3) or FlyBase [7] (Figure 4). Hence, it is possible to view population genetic data together with annotation, sequence conservation and gene expression data (Figure 5). Users interested in the SNPs in their region of interest can display a table of polymorphic sites (Figure 6), providing the position, alleles and their frequency in the population. In coding regions also polymorphic amino acids are displayed. Users interested in the indels in their region of interest can display a table of insertion deletion sites (Figure 7), providing the position, and indel sequences in the population.

### Population Variation Parameters and Tajima's D

Natural variation is typically measured by pairwise sequence comparisons (Tajima's π [9]) or the number of segregating sites in a sample of DNA sequences (Watterson's *θ *[10]). Under neutrality and constant population sizes both estimators are unbiased. As selection and demographic events affect both estimators differently, the weighted contrast between Tajima's π and Watterson's *θ *(Tajima's D [11]) is frequently used to infer selection and/or population size changes. For any genomic region of interest, users can infer the two population genetic variation parameters Watterson's *θ *or Tajima's *π *as well as Tajimas' D [4] (Figure 3, Figure 4, Figure 5). As each of these measurements is calculated with sliding windows over the specified genomic region, the user can define parameters thresholds describing properties of the windows and of the data within the window. Furthermore, users can define whether they are interested in calculating the specified measure in the regions flanking the fragment of interest, e.g. 100 bp at the 5' end and 3' end gene CG2714 (default: 0 bp). PoPoolation DB provides the user with Watterson's *θ*, Tajima's *π *and Tajima's D values pre-calculated using the default parameters.

Currently there is a limitation to the size of the fragment that can be queried, i.e. 100.000 bp. PoPoolation DB also provides 95% and 99% quantiles for each population genetic estimator and chromosome. For computational efficiency, Popoolation DB does not recalculate the quantiles each time a fragment is queried. Rather pre-calculated quantiles for window sizes in a range between 500 bp and 50.000 bp are stored in the database. PoPoolation DB automatically displays the quantiles for the window size closest to the user defined one.

The following is a list and a description of the window parameter thresholds:

Window Size: Length in bp of the window within which the measurement will be calculated (default: 1.000 bp)

Step Size: Number of bp that the window should be moved along the chromosome, e.g. for windows of 1000 bp a step size of 100 bp implies that two neighboring windows overlap in 900 bp (default: 100 bp).

Minimum Count: Every identified SNP requires at least two alleles where each allele has to occur at least 'Minimum Count' times (default: 2).

Minimum Quality Score: Minimum base quality required for a base to be considered in the analysis (default: 20).

Minimum Coverage: Minimum coverage threshold, below which the measurement is not calculated and no SNPs will be identified (default: 4).

Maximum Coverage: Maximum coverage threshold above which the measurement is not calculated and no SNPs will be identified (default: 300).

Minimum Covered Fraction: Proportion of the window that should have sufficient coverage, i.e: larger or equal than minimum coverage and smaller or equal than maximum coverage (default: 0.6).

### SNP and Codon Information

PoPoolation DB provides SNP and codon information for the genomic region of interest in display and download mode. The table can be downloaded in a tabular format. For each position in the queried region PoPoolation DB prints the annotated features available for the fragment (e.g. introns, CDS) as stored in gff3 files in Flybase. It also provides a hyperlink to the corresponding gene in which the feature is located in Flybase.

For each SNP in the region of interest, allele counts, coverage and the character state in the *D. melanogaster *reference genome are provided (Figure 6). The SNP table also contains information about the amino acid in the reference genome and whether a polymorphism is silent or alters the amino acid (e.g. Position X-2582776: Reference codon GAT: Reference amino acid Asp; Variant codon TAT; Variant amino acid Tyr).

### Indel Information

PoPoolation DB also prints a table with the indel information of the region of interest (Figure 7). The table can be downloaded in tabular format. For each reference position having an indel, PoPoolation DB shows the frequency of the indel and the nucleotide sequence that is added or deleted. Deletions are marked with a minus (e.g -3AGG) and insertions with a plus (e.g. +4ATCG). For positions with complex indels (e.g.: several different insertions at the same reference position), PoPoolation DB prints separate rows for each type of change (e.g. rows 10 to 12 in Figure 7). The position of indels refers to one position before the indel in the reference sequence. The coverage of the indel is provided as the average of the five neighboring nucleotides on each side of the indel.

### Evaluation of the database

PoPoolation DB has been designed to provide maximum flexibility for the user. This requires calculating the population genetic parameters for every query separately. Depending mainly on the maximum coverage and the size of the genomic region considered, the calculations may be time consuming due to the need of adjusting the calculations for each site due to the heterogeneity in coverage. Table 1 contains some benchmarks about the expected query times.

**Table 1:** Benchmarks for processing time of PoPoolationDB

Size of query	Average processing time (minutes)
10 kb	3
50 kb	6.50
100 kb	10.50

Average processing time was calculated from 10 randomly selected genomic regions with the following parameters: minimum count: 2, minimum coverage: 4, maximum coverage: 500, window size:1 kb

### Reproducibility

In order to increase transparency and reproducibility of the results, PoPoolation DB prints for every query a log file with the parameters used. Additionally PoPoolation DB offers to download all information (SNP and codon table, Indel table) produced by it, which will be especially useful for further downstream analysis. PoPoolation DB not only visualizes the population genetic estimators track in the UCSC and Flybase genome and RNA-Seq browsers, but also allows downloading in the widely used Wiggle file format.

### Limitations

PoPoolation DB is restricted to the analysis of Illumina sequence reads generated from pooled DNA samples. Currently, it is not possible to load sequence data obtained from sequencing individuals separately, as this requires an entirely different handling of data.

### Future Directions

The current version of PoPoolation DB has the polymorphism information for one population of *D. melanogaster*. We plan to add more populations from *Drosophila melanogaster *and *D. simulans*. Moreover, we will integrate tools to compare the polymorphism data from various populations.

## Conclusions

PoPoolation DB is a user friendly integrated database. This database allows the retrieval of polymorphism data from pooled 2nd generation sequencing data using new statistical approaches to obtain population genetic parameters from pooled data. PoPoolation DB will enable researchers to identify natural polymorphism, their frequencies, and associated functional annotations from UCSC and Flybase genome browsers. Furthermore, population genetic estimators and RNA-Seq information can be obtained for a genomic region of interest.

We anticipate that the database will not only be of interest for the identification of segregating functional variants, but also facilitate primer design and comparative analyses. Currently, PoPoolation DB provides polymorphism data from a single D. *melanogaster *population from Portugal, but additional populations and species will be uploaded as soon as they become publicly available.

## Availability & requirements

PoPoolation DB is freely available to all non-commercial users at http://www.popoolation.at/pgt

## Authors' contributions

CS designed the project. RVP designed the database and web interface, and integrated the data. PO and RVP wrote the manuscript. PO generated pileup data. RK wrote the program to calculate population genetic estimators. RK, VN and PO did database testing. VN generated the sequencing data. CS revised the manuscript. All authors read and approved the final manuscript.
